# Epigenetic marks or not? The discovery of novel DNA modifications in eukaryotes

**DOI:** 10.1016/j.jbc.2024.106791

**Published:** 2024-02-23

**Authors:** Wei-Ying Meng, Zi-Xin Wang, Yunfang Zhang, Yujun Hou, Jian-Huang Xue

**Affiliations:** 1 Key Laboratory of Spine and Spinal Cord Injury Repair and Regeneration of Ministry of Education, Tongji Hospital affiliated to Tongji University, Frontier Science Center for Stem Cell Research, School of Life Sciences and Technology, Tongji University, Shanghai, China; 2 Clinical and Translational Research Center of Shanghai First Maternity and Infant Hospital, Shanghai Key Laboratory of Signaling and Disease Research, Frontier Science Center for Stem Cell Research, School of Life Sciences and Technology, Tongji University, Shanghai, China; 3 Institute for Regenerative Medicine, Shanghai East Hospital, Shanghai Key Laboratory of Signaling and Disease Research, Frontier Science Center for Stem Cell Research, School of Life Sciences and Technology, Tongji University, Shanghai, China

**Keywords:** epigenetic marks, DNA modifications, DNA methylation, DNA demethylation, TET dioxygenases, transcriptional regulation, eukaryotes, 5-methylcytosine (5mC), 5-hydroxymethylcytosine (5hmC), *N*^*6*^-methyladenosine (6mA)

## Abstract

DNA modifications add another layer of complexity to the eukaryotic genome to regulate gene expression, playing critical roles as epigenetic marks. In eukaryotes, the study of DNA epigenetic modifications has been confined to 5mC and its derivatives for decades. However, rapid developing approaches have witnessed the expansion of DNA modification reservoirs during the past several years, including the identification of 6mA, 5gmC, 4mC, and 4acC in diverse organisms. However, whether these DNA modifications function as epigenetic marks requires careful consideration. In this review, we try to present a panorama of all the DNA epigenetic modifications in eukaryotes, emphasizing recent breakthroughs in the identification of novel DNA modifications. The characterization of their roles in transcriptional regulation as potential epigenetic marks is summarized. More importantly, the pathways for generating or eliminating these DNA modifications, as well as the proteins involved are comprehensively dissected. Furthermore, we briefly discuss the potential challenges and perspectives, which should be taken into account while investigating novel DNA modifications.

The genomic DNA of most living organisms is composed of four basic nucleotides, which could be further modified to increase the versatility of DNA by altering its chemical properties. DNA modification can be triggered by a series of enzymes or induced spontaneously by DNA damage or metabolites (1, 2). But in general, only enzyme-mediated DNA modifications are regarded as “epigenetic marks” for their ability to faithfully transmit these parental imprints into daughter cells after cell division. Additionally, DNA epigenetic modifications normally undergo dynamic changes to orchestrate various biological processes during development, which are synergistically regulated by the “writer” and “eraser” proteins. Although many kinds of DNA modifications have been detected, they are hardly recognized as “epigenetic marks” before the related writers or erasers are identified. In this review, we only focus on the DNA modifications that are catalyzed by enzymes.

With the advancements in detection methodologies, a wide range of DNA modifications have been identified across diverse organisms. To date, a minimum of 11 types of DNA modifications have been documented as potential epigenetic marks in eukaryotes (Fig. 1). All these DNA modifications take place at the nucleotide bases, without disrupting their ability to pair with complementary bases. Methylation is the most prevalent form of DNA modifications, spread widely throughout nearly all forms of life (3). DNA methylation can occur at either the N4 or C5 position of cytosines, termed *N*^*4*^-methylcytosine (4mC) and 5-methylcytosine (5mC) (4), respectively. Adenosines can also be methylated into *N*^*6*^-methyladenosines (6mA) (5). While 5mC has been found to be abundant in eukaryotes including mammals, 4mC and 6mA are dominant in prokaryotes. Hydroxymethylation is another form of modification initially identified at the methyl group of thymidines (5-hydroxymethyluridine, 5hmU). In the genome of kinetoplastids, 5hmU could be further modified into β-D-glucosyl-hydroxymethyluridine (Base J), which is the first hypermodified base found in eukaryotic DNA (6). In the search for the homologs of J binding protein 1/2 (JBP1 and JBP2 in human cells, which are required for 5hmU biosynthesis and belong to 2-oxoglutarate (2-OG) and Fe(II)-dependent oxygenase superfamily, three novel proteins were identified: TET1, TET2, and TET3. Ten-eleven translocation (TET) proteins are responsible for the iterative oxidation of 5mC into 5-hydroxymethylcytosine (5hmC), 5-formylcytosine (5fC), and 5-carboxylcytosine (5caC) (7). Although TET proteins exhibit similar activities across various genomes, a unique TET-homolog named 5mC modifying enzyme 1 (CMD1) in green algae, has been observed to convert 5mC into 5-glyceryl-methylcytosine (5gmC) (8). In addition, a novel cytosine modification known as *N*^*4*^-acetyldeoxycytosine (4acC), previously detected in RNA, has now been discovered in the genomic DNA of *Arabidopsis* (9). Lastly, a 5hmC-like modification in DNA has been found in the human malaria parasite (10), but its molecular structure still requires further determination.

**Figure 1:**
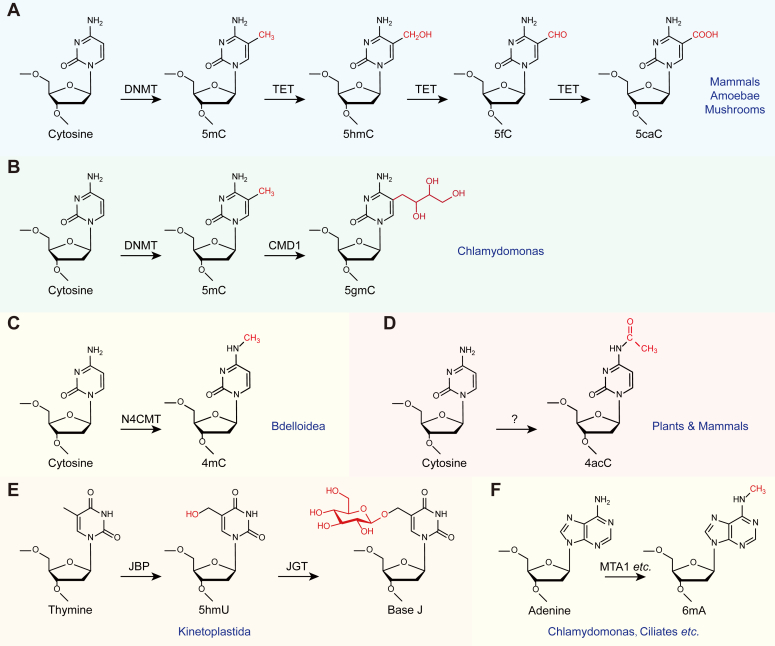
**Eukaryotic DNA modifications in different nucleotides.***A*, in mammals, 5mC is generated from cytosines by DNMT, which is subjected to TET-mediated oxidation, resulting in the formation of 5hmC, 5fC, and 5caC. *B*, 5mC is converted into 5gmC by CMD1 in *Chlamydomonas reinhardtii*. *C*, in *Bdelloid rotifers*, the generation of 4mC is facilitated by N4CMT. *D*, 4acC, modified by unidentified enzymes, was detected in various plants and mammals. *E*, in the majority of the *Kinetoplastida*, the conversion of thymine to 5hmU is attributed to the enzymes JBP1/2. 5hmU is subsequently hypermodified by JGT, resulting in the formation of base J. *F*, 6mA has been reported to be present in numerous organisms, and the MTA1 complex has been identified as the authentic 6mA methyltransferase in ciliates. 4acC, *N*^*4*^-acetyldeoxycytosine; 5caC, 5-carboxylcytosine; 5fC, 5-formylcytosine; 5mC, 5-methylcytosine; 5hmC, 5-hydroxymethylcytosine; 5hmU, 5-hydroxymethyluridine; 6mA, *N*^*6*^-methyladenosine; CMD1, 5mC modifying enzyme 1; DNMT, DNA methyltransferase; JBP, J binding protein; JGT, base J–associated glucosyltransferase; MTA1, 6mA methyltransferase; N4CMT, 4mC methyltransferase; TET, ten-eleven translocation.

In this review, we aim to provide a comprehensive overview of the latest advancements in the studies of canonical DNA methylation and the discovery of novel DNA modifications in eukaryotes. The enzymes or pathways involved in the dynamic change of each DNA modification are dissected, along with the characterization of their functions and molecular mechanisms. Finally, we discuss the potential challenges and pitfalls associated with the identification and quantification of novel DNA modifications.

## DNA modifications on thymidines

### β-D-glucosyl-hydroxymethyluridine and 5hmU

Base J was identified in the nuclear DNA of *Trypanosoma brucei* in 1993 and subsequently detected in all kinetoplastids as well as certain unicellular algae that are close to the *Kinetoplastida*. Base J seems to be absent in other protozoa or metazoa, including mammalian cells (6). The biosynthesis of J involves a two-step process, and J represents the first hypermodified base found in eukaryotic DNA. Firstly, the thymidines are converted into 5hmU, catalyzed by JBP1 and JBP2 proteins (11, 12). 5hmU is then subjected to glycosylation by the base J–associated glucosyltransferase (JGT), resulting in the formation of base J (13). Base J constitutes approximately 0.5% to 1% of thymidines in the genome of *T. brucei*. In contrast, 5hmU only replaces nearly 0.01% of total thymidines and accounts for approximately 2% of base J in *T. brucei*, implicating a substantial enzymatic activity of JGT in 5hmU turnover (6, 14). In *Leishmania*, it was observed that 90% of 5hmU-enriched loci overlapped with J (15). However, as an intermediate to form base J, whether 5hmU functions as a specific epigenetic mark remains uncertain.

Base J is presumably related to gene repression as it is exceptionally identified in the inactivated variant surface glycoprotein expression sites in the bloodstream form of *T. brucei* (6). This resembles the suppressive role of 5mC in vertebrates and plants, which is rarely detected in *T. brucei* DNA (16). However, the strain depleted of both JBP1 and JBP2 survived well, but the expression sites were not reactivated, suggesting that the occurrence of base J at these regions should be a consequence of gene repression but not the cause (6). Nevertheless, base J can function as an epigenetic mark undoubtedly since the enrichment of J in telomeric repeats was found in nearly all the kinetoplastid flagellates (17). This strongly indicates the involvement of J in telomere regulation but the mechanisms are still controversial. Moreover, in *Leishmania*, about 1% of base J is located at chromosome-internal RNA polymerase II termination sites, which were shown to be essential for proper transcription termination. The massive readthrough of transcriptional stops induced by J depletion would lead to the lethality of *Leishmania* (18). Another study also presented evidence for the regulation of Pol II transcription initiation *via* base J and chromatin remodeling in kinetoplastids (19).

The enzymes responsible for the generation of base J have been identified. JBP1 and JBP2 are two thymidine hydroxylases (THs) that belong to Fe(II)- and 2-OG–dependent dioxygenase superfamily. They share a conserved TH domain in the N-terminal region. The TH domain is characterized by a His-X-Asp (HxD) motif, which is essential for Fe(II) binding. This domain also contains an Arg residue critical for the binding of 2-OG. Mutation at these specific sites completely abrogates the formation of 5hmU *in vivo* (11, 20). In the C-terminal region, JBP1 contains a J-binding domain, whereas JBP2 is composed of a SWI2/SNF2 domain, indicating a distinct function between JBP1 and JBP2. A simplified model has been proposed (6) in which JBP2 is responsible for the *de novo* synthesis of base J and also the localization of base J to the right sites in the genome. Subsequently, JBP1 will bind to the base J *via* its DNA-binding domain and generate more J in the flanking loci or catalyze the formation of J in the newly synthesized DNA strand, following DNA replication, reminiscent of the roles of mammalian DNA methyltransferase (DNMT) family proteins in DNA methylation (21). Moreover, another study showed that JBP1 also possesses *de novo* J-synthesis activity at chromosome-internal regions (22). Most interestingly, JBP-like proteins were found to be conserved in many organisms. The study of JBP family proteins facilitates the discovery of several cytosine oxygenases, which will be discussed below.

Another enzyme related to base J is JGT, which was identified to catalyze the transfer of glucose from uridine diphosphoglucose to 5hmU within the context of dsDNA (13). JGT was confirmed as the only glucosyltransferase responsible for J formation as the deletion of JGT in *T. brucei* resulted in a cell line that completely lacked base J (13). In contrast to JBP, JGT homologs in metazoa were predicted to be catalytic inactive, consistent with the undetectable level of base J in these organisms (23). Unfortunately, the proteins responsible for eliminating base J and 5hmU still lack in-depth study, which is important for understanding the functions of these DNA modifications as epigenetic marks in *Kinetoplastida*.

## DNA modifications on cytidines

### 5-Methylcytosine

5mC was initially detected in bacteria in 1925 and mammals in 1948 (3). The research of 5mC is majorly focused on vertebrates and plants during the past 50 years. In mammalian cells, 5mC occurs predominantly at palindromic CpG dinucleotides while rarely detected at CpH or CHH contexts (H refers to A/C/T). Around 70% of the CpG sites are methylated in mammalian somatic genomes (24). Interestingly, 5mC only accounts for approximately 3% of the total cytosines (25) due to the loss of CpG in the genome which might result from CpG methylation or the inherent mutability during evolution (26). 5mC has been detected in numerous species, encompassing a wide range of mammals, plants, and lower eukaryotes. Apart from mammals, 5mC was found at both CpG and non-CpG sites in plants and other eukaryotes (8, 27, 28). Intriguingly, 5mC seems absent in two commonly used model systems: *Drosophila melanogaster* and *Saccharomyces cerevisiae* (28). In light of the significant roles that DNA methylation plays in transcriptional regulation, an alternative epigenetic regulatory pathway might be utilized such as other forms of DNA modifications (*e.g.*, 6mA) or histone modifications in these organisms.

5mC plays crucial roles in the spatiotemporal control of gene expression and various biological processes such as genomic imprinting, X chromosome inactivation, and transposon suppression in mammals (29, 30). Genome-wide 5mC landscape revealed that 5mC resides prevalently in repetitive elements, playing a significant role in the silencing of transposons (31). In contrast, many CpG islands (CpG-rich genomic regions) within the promoter regions were found to be hypomethylated. However, the expression level of genes with CpG-rich genomic regions promoter was sensitive to DNA methylation (32). 5mC is normally linked to transcriptional suppression due to reduced binding affinity of transcription factors with methylated DNA (especially enhancer regions). Moreover, 5mC could recruit a category of reader proteins harboring methyl-CpG-binding domain (MBD), including methyl CpG binding protein 2 (MeCP2) and methyl-CpG-binding domain 1. The incorporation of MBD proteins repels the transcription factors from promoters, thereby impeding gene expression. Nevertheless, the presence of 5mC within gene bodies has been found to be correlated with increased gene expression, potentially attributed to the inhibition of alternative transcription initiation (24, 33). In plants, 5mC is observed to be enriched in transposon-rich heterochromatin, also implicating a suppressive role in gene expression (27). However, SUVH1 and SUVH3 were unexpectedly found to bind methylated DNA and formed a complex with DNAJ domain–containing proteins in *Arabidopsis*. The involvement of DNAJ proteins enhances gene transcription in both plants and mammals (34), suggesting a more complicated role of DNA methylation in transcriptional regulation.

As the best-studied DNA epigenetic mark, the formation of 5mC has been extensively studied. In mammals, 5mC is generated by DNMTs *via* the transfer of methyl groups to cytosines from SAM. The DNMT family is composed of at least six members. DNMT3A and DNMT3B are responsible for *de novo* methylation of cytosines, whereas DNMT1 is recruited by ubiquitin-like PHD and RING finger domain-containing protein 1 (UHRF1) to replication forks in order to maintain the methylation pattern during DNA replication in the S phase (35). DNMT3C was only reported in rodents and responsible for methylation of evolutionarily young retrotransposons (36). In contrast, DNMT3L is inactive due to the partial deletion of its methyltransferase motif. Instead, DNMT3L was found to be a critical cofactor in enhancing the enzymatic activity of DNMT3A/3B *via* forming a butterfly-shaped heterotetramer (37, 38).

DNMT proteins are not enough for the accomplishment of DNA methylation in mammals. Especially for *de novo* methylation, one remaining question is how the DNMTs are directed to the right position. The crosstalk between DNA methylation and histone modifications or RNA methylation provides one possibility. DNMT3L interacts with histone H3 tails, which is significantly inhibited by methylation at lysine 4 of H3 (H3K4me3) (39). Further study showed the ADD domain of DNMT3A also interacts with H3 and nucleosomes, while H3K4me3 disrupts the releasement of autoinhibition of DNMT3A induced by H3 tails (40, 41). In addition, the methylation at lysine 36 of H3 (H3K36me2 and H3K36me3) binds with the PWWP domain of DNMT3A/3B (42, 43), whereas H3.3G34R mutation severely compromised the interaction (44). It is believed that other histone epigenetic marks will also contribute to the *de novo* methylation process. Furthermore, RNA *N*^*6*^-methyladenosine (m6A) was shown to regulate DNA demethylation and chromatin accessibility, which could also affect the deposition of 5mC in the genome (45, 46).

The generation of 5mC involves several DNA methyltransferases in plants. Domain rearranged methyltransferase 2, a plant homolog of DNMT3, has been demonstrated to catalyze *de novo* DNA methylation. Unlike that in mammals, the *de novo* DNA methylation in plants requires 24-nt siRNA to recruit methyltransferases, known as RNA-directed DNA methylation. Plant DNA methylation is maintained by additional methyltransferases including MET1 (DNA methyltransferase 1) at CpG sites and chromomethylase 2/3 (CMT2/3) at CHG sites. Notably, CHH sites can only be *de novo* methylated by domain rearranged methyltransferase 2 or CMT2 since it is not a palindromic context (27).

**Figure 2:**
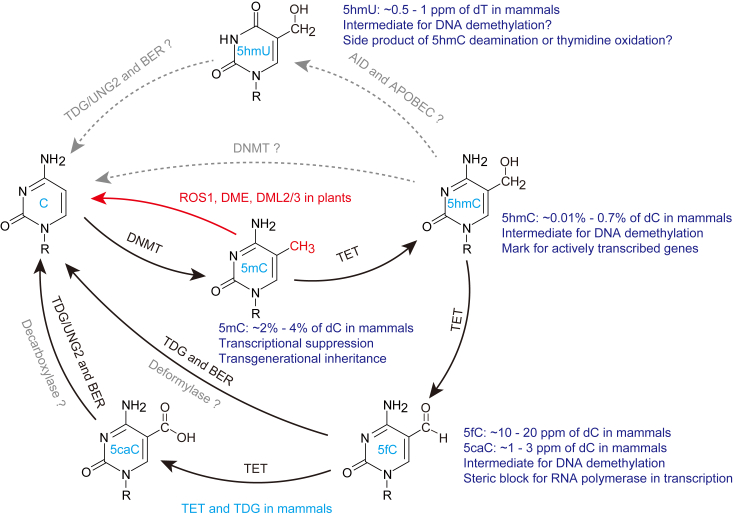
**Active DNA demethylation pathways in plants and mammals.** In plants, ROS1 and its homologs cleave 5mC bases, followed by the BER pathway to restore unmodified cytosines, leading to active DNA demethylation. In mammalian cells, TET-mediated DNA oxidation, along with the TDG-initiated BER pathway, constitutes a major pathway in active DNA demethylation. In addition, 5hmC, 5fC, and 5caC might also function as independent epigenetic marks in mammals. 5caC, 5-carboxylcytosine; 5fC, 5-formylcytosine; 5hmC, 5-hydroxymethylcytosine; BER, base excision repair; ROS1, repressor of silencing 1; TDG, thymine DNA glycosylase, TET, ten-eleven translocation.

5mC is reversible and dynamically regulated both in mammals and plants (47), which is a hallmark of epigenetic marks. In general, the DNA methylation pattern is maintained after cell division. Loss or decreased activity of the maintenance machinery will result in the dilution of 5mC after DNA replication, which is demonstrated as passive DNA demethylation. By contrast, active DNA demethylation refers to the enzymatic elimination of methyl groups, or 5mC bases, or even nucleotides from the genome (48) (Fig. 2). As of recently, no demethylase that can wipe off the methyl groups directly from 5mC has been discovered. Repressor of silencing 1, the first DNA “demethylase” identified in *Arabidopsis thaliana*, was found to be a 5mC-specific glycosylase. Repressor of silencing 1 and its homologs (DME; DML2/3) are responsible for the cleavage of 5mC bases, resulting in the formation of apyrimidinic sites, which are repaired through the subsequent base excision repair (BER) pathway, leading to the restoration of the unmethylated cytosines (27, 49).

Although 11 DNA glycosylases were found in human and mouse cells, only methyl-CpG binding domain 4 and thymine DNA glycosylase (TDG) show very weak cleavage activity against 5mC *in vitro*. Alternatively, activation induced cytidine deaminase or apolipoprotein B mRNA editing enzyme catalytic subunit-catalyzed 5mC deamination followed by MBD4/TDG-mediated cleavage of G:T mismatch was proposed as a potential mechanism for DNA demethylation, but no direct evidence for activation induced cytidine deaminase- or apolipoprotein B mRNA editing enzyme catalytic subunit-triggered 5mC deamination was observed *in vitro* or *in vivo* within dsDNA (48, 50). A break in this field did not come until the characterization of TET dioxygenases, which convert 5mC into 5hmC, 5fC, and 5caC successively. The latter two are subjected to TDG-mediated excision and BER, thus achieving DNA demethylation (25, 51, 52). Moreover, the oxidized 5mC destroys the recognition of hemi-methylated DNA by UHRF1 during maintenance DNA methylation, resulting in passive DNA demethylation (53). The mechanisms underlying active DNA demethylation in mammalian cells are still controversial. Actually, two distinct waves of global DNA methylation reprogramming happen during mammalian development, and active DNA demethylation was shown to take place in zygotes (24). It is still under debate whether active DNA demethylation in zygotes is associated with TET-mediated DNA hydroxymethylation (54, 55, 56). Since TDG was shown to be dispensable for zygotic DNA demethylation (56), uracil DNA glycosylase 2 and Nei like DNA glycosylase 3 glycosylases were also indicated to be associated with 5caC-mediated DNA demethylation (57, 58). Despite that DNA repair might constitute an important part of active DNA demethylation at specific loci, it is imperative to identify and characterize alternative pathways that exhibit a higher efficacy with a lower risk of chromosome instability in genome-wide DNA demethylation (59, 60).

### 5-Hydroxymethylcytosine, 5-formylcytosine, and 5-carboxylcytosine

The widespread distribution of 5hmC in mammalian cells and tissues was initially reported in 2009, revealing a specific enrichment of 5hmC in Purkinje neurons in the brain (61). A simultaneous work demonstrated that 5hmC was generated by TET dioxygenases, which are JBP homologs in both human and mouse cells (51). Subsequent studies advanced our understanding by demonstrating that 5hmC could be further converted into 5fC and 5caC by TET proteins (25, 52), albeit the activity is comparatively lower than using 5mC as the substrate (62). In embryonic stem (ES) cells, 5hmC accounts for approximately 0.1% (1000 ppm) of total cytosines, while 5fC and 5caC only constitute about 20 ppm and 3 ppm of total cytosines, respectively. In the cerebral cortex, 5hmC is specifically enriched to a level of approximately 0.7% of total cytosines, accounting for 20% of 5mC. However, 5fC and 5caC do not show significant enrichment, implicating a specific role of 5hmC as an epigenetic mark in the brain (25).

Nevertheless, due to the low abundance of 5hmC and trace amounts of 5fC and 5caC within the genome, it is still disputable whether these 5mC-derived DNA modifications can be regarded as epigenetic marks and the functions of 5hmC, 5fC, and 5caC remain largely unexplored. However, at least one critical role of these 5mC derivatives is to serve as intermediates in the process of active and passive DNA demethylation (Fig. 2). During maintenance DNA methylation, UHRF1 binds specifically to hemimethylated DNA and facilitates the recruitment of DNMT1 to targeted sites. The binding affinity is abrogated when 5mC is converted into 5hmC or other derivatives, thus leading to the inaccessibility of DNMT1 (50). Furthermore, 5fC and 5caC are suitable substrates for TDG and potentially other glycosylases. The elimination of these modified bases followed by BER is a confident route to achieve active DNA demethylation, which has been demonstrated in different cell types (63). Interestingly, the mammalian and bacterial DNMTs have been shown to possess the activity to remove the methyl groups, the hydroxymethyl groups, or the carboxyl groups directly from 5mC, 5hmC, or 5caC *in vitro*, respectively (64, 65, 66). Nevertheless, it is controversial to confirm the bifunctional activity of DNMT *in vivo*. Using isotope-labeled or fluorinated DNA, the deformylation of 5fC and the decarboxylation of 5caC were observed within the cells (59, 67). However, the enzymes capable of C-C bond cleavage are yet to be demonstrated in mammals. One possible clue arises from the isoorotate decarboxylase in the T-salvage pathway that catalyzes the decarboxylation of 5caU to U in fungi. Isoorotate decarboxylase was also shown to exhibit weak activity against 5caC (68), implicating the existence of homologous enzymes responsible for the decarboxylation of 5caC in higher organisms.

Meanwhile, evidence has been presented to support the role of 5hmC as a stable epigenetic mark. Similar to 5mC, the functions of 5hmC and its derivatives partially depend on their reader proteins. Numerous potential readers have been identified for 5hmC, 5fC, and 5caC in different cells, but the specific roles of these proteins remain largely unexplored (53). Consistently, a large number of DNA repair proteins in mouse ES cells were identified, supporting the involvement of DNA repair in active DNA demethylation. Interestingly, UHRF2, rather than UHRF1, has been identified as a 5hmC-specific reader. UHRF2 is allosterically activated by 5hmC to exert its E3 ligase activity, facilitating the process of active DNA demethylation (53, 69). Moreover, 5hmC has been reported to be enriched within the gene body of actively transcribed genes, positively correlated with gene expression (70, 71). MeCP2, a conventional 5mC-binding protein, also binds with 5hmC, especially for hydroxymethylated CpA repeats. Loss of MeCP2 results in nucleosome invasion and transcriptional dysregulation (72). On the contrary, the presence of 5fC and 5caC forms specific hydrogen bonds between the formyl or carboxyl group and the conserved epi-DNA recognition loop of the RNA polymerase II, causing a steric block for incoming nucleotides, thus reducing the elongation rate of Pol II during transcription (73, 74). However, the evidence to support 5fC and 5caC as epigenetic marks is still weak, which requires in-depth determination.

**Figure 3:**
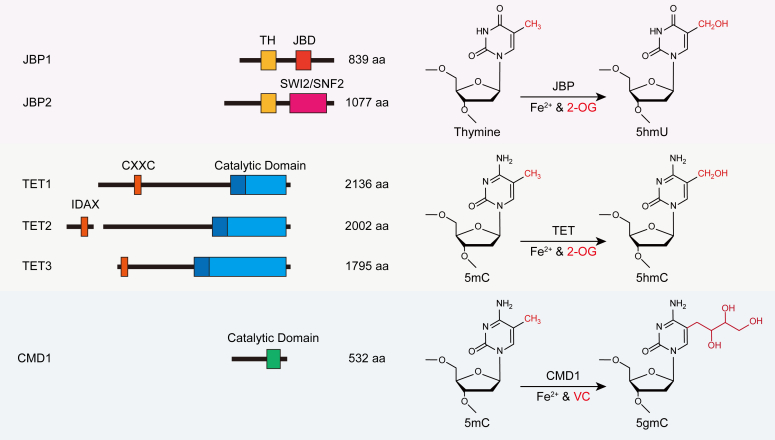
**The schematic diagrams of different TET_JBP domain–containing oxygenases and their enzymatic activities.** TET, ten-eleven translocation; JBP, J binding protein.

The TET dioxygenase is the only enzyme responsible for the generation of 5hmC, 5fC, and 5caC in mammalian cells. The TET family comprises three members in human and mouse cells: TET1, TET2, and TET3 (Fig. 3). TET proteins are conserved in the C-terminal catalytic domain, which adopts a double-stranded β-helix fold. The double-stranded β-helix fold is highly conserved at the HxD motif, which chelates with Fe(II) in most of the dioxygenases including TET and JBP. The binding sites of 2-OG are more dynamic in dioxygenases, whereas in TET proteins, at least two conserved Arg residues are utilized. The C-terminal catalytic domain is enough to elicit dioxygenase activity *in vitro* (75). This catalytic region contains an intrinsically disordered region, which shows autoinhibitory effects for the dioxygenase activity (76). TET proteins exhibit a higher level of complexity due to the presence of multiple additional domains. A N-terminal CXXC domain exists in TET1 and TET3. The inverted CXXC domain of TET2 detached from TET2 and formed another protein named IDAX. Both CXXC and IDAX are also involved in the autoinhibitory effect for TET proteins (7, 50, 77). TET proteins are regulated by multiple cofactors. 2-OG, generated from isocitrate by IDH1 and IDH2 (isocitrate dehydrogenase) in mammals, is crucial for the enzymatic activity of TET proteins (78). Gain-of-function mutations in *IDH1/2* might result in a similar phenotype to that of *TET2* mutants (79). Ascorbic acid (also known as vitamin C, VC) serves as another cofactor required for enhancing TET-mediated reactions (47, 80). Furthermore, the level of oxygen also influences the activity of TET dioxygenases (81). Nevertheless, these cofactors have a broad effect on a range of dioxygenases including RNA demethylases and histone demethylases, implicating critical crosstalk between epigenetics and small-molecule metabolites. Functionally, TET family proteins are redundant but play specific indispensable roles exclusively within a particular environment. For instance, the expression level of *TET1* and *TET2* is elevated in ES cells with *TET3* exhibiting higher expression specifically in oocytes and zygotes (82). On the other hand, TET2 cooperates with TET3 during hematopoiesis (83). *TET2* mutations are frequently observed in leukemia (84). However, *TET1* expression was reported to be upregulated in acute myeloid leukemia, suggesting an opposite role of TET1 and TET2 during leukemogenesis (85).

TET proteins are widely distributed across the kingdoms of life (86), and the 5mC derivatives are also detected by the dioxygenase assay *in vitro* using TET proteins from ameboflagellate (87) and mushrooms (88). However, the presence and functions of 5hmC and its derivatives in these organisms remain unexplored. Interestingly, like JBP proteins, TET could also convert thymine into 5hmU in mouse ES cells, at a level similar to 5caC (89). It is unclear whether 5hmU plays a unique role as an epigenetic mark or merely as a side product of TET-catalyzed oxidation in mammals.

Recently, most work that studied the function of 5mC derivatives relies on the manipulation of *TET* genes. In this case, it is not easy to distinguish the function of TET proteins from DNA modifications. Actually, TET proteins can also function through their noncatalytic roles in chromatin remodeling or transcriptional regulation (90, 91). Although a specific mutation of the HxD motif in TET proteins can be generated, it is still difficult to pinpoint the particular roles of 5hmC, 5fC, and 5caC. Interestingly, a single mutation in Thr1372 of human TET2 could stall the oxidation mostly in 5hmC, which might be useful for studying the function of 5hmC (92). An alternative approach is to characterize the specific readers for each modified nucleotide, which also needs further elaborated studies due to the relatively lower abundance of 5mC derivatives.

### 5-Glyceryl-methylcytosine

TET_JBP proteins have been reported to be present in various organisms including green algae (86), with the enzymatic activity in most species remaining unexplored. A TET homologue named CMD1 in the model system *Chlamydomonas reinhardtii* was unexpectedly identified to have a unique activity to convert 5mC into 5gmC (Fig. 1). Instead of 2-OG, CMD1 utilizes VC as a cosubstrate and catalyzes the direct transfer of the C4-C6 moiety of VC to the methyl group of 5mC, resulting in the formation of 5gmC with glyoxylate and carbon dioxide as byproducts (8). 5gmC is another type of DNA modification that was found to be hypermodified directly by metabolites other than base J, inferring a complicated role of VC in transcriptional control beyond its canonical function in adjusting redox reaction. Compared with other DNA modifications, 5gmC is significantly larger. It is unclear whether 5gmC will serve as a steric block for transcription like 5caC, even though the 5gmC:G pairing remains unaffected.

In *C. reinhardtii*, the abundance of 5gmC is approximately 10 ppm of total cytosines, comparable to the level of 5fC in ES cells (8). Further investigation is required to determine whether 5gmC could serve as an independent epigenetic mark *in vivo*, such as to identify the reader proteins for 5gmC or to profile the global landscape of 5gmC in the genome. Like 5hmC and its derivatives, 5gmC can also serve as an intermediate to promote DNA demethylation, which is critical for the transcriptional control of *LHCSR3* in photoacclimation in green algae (8). Our preliminary data show the significant enrichment of 5gmC at CpG sites, which is consistent with the differentially methylated regions observed after *CMD1* depletion (8). As 5mC is evenly distributed in CpG and non-CpG sites in *C. reinhardtii* (93), the unique distribution pattern of 5gmC might implicate its role as a stable epigenetic mark. One remaining question is how 5gmC is actively removed. Although several DNA glycosylases exist in *C. reinhardtii*, their activity to remove 5gmC or even 5mC requires in-depth determination.

The HxD motif in dioxygenases, which coordinates with Fe(II), is also conserved in the catalytic domain of CMD1 (Fig. 3). However, the canonical 2-OG binding sites are absent in CMD1, consistent with the dispensable role of 2-OG in the CMD1 reaction. According to the structure of CMD1, VC forms several direct or indirect hydrogen bonds with its adjacent residues, but the *in vitro* assay revealed a weak interaction between VC and CMD1 (94). In the CMD1 reaction, the lactone form of VC mono-coordinates Fe(II) in a manner different from 2-OG (94). CMD1 exhibits moderate substrate preference for CpG sites, in consistency with the distribution pattern of 5gmC (8). CMD1 has at least seven other homologs in *C. reinhardtii* and the CMD1-like proteins are widespread in the close relatives of green algae. However, the existence of 5gmC and the enzymatic activity of CMD1-like proteins in other organisms require further study. Moreover, three distinct types of TET_JBP domain–containing oxygenases have revealed diverse activities, which involve the utilization of various substrates and cofactors (Fig. 3). It is interesting to find out how these oxygenases distribute and evolve within the phylogenic tree. Whether other kinds of TET_JBP proteins exist, potentially using different cofactors for various substrates remains an open question.

**Figure 4:**
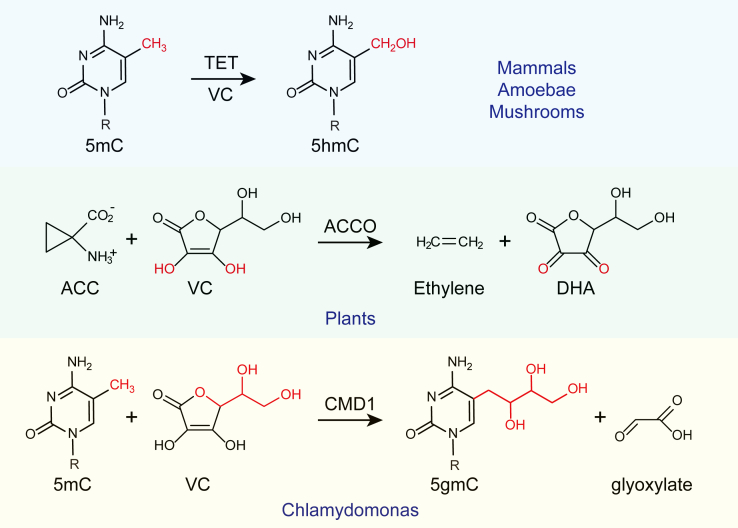
**The multifaceted roles of VC in non-heme iron-dependent oxygenases.** VC, vitamin C.

The CMD1-mediated reaction is a newly characterized reaction using VC as a cosubstrate. For the majority of oxygenases, VC functions to enhance the enzymatic activity without contributing itself to the reaction. One notable exception pertains to the reaction catalyzed by 1-aminocyclopropane-1-carboxylic acid oxidase (ACCO), which is responsible for the formation of ethylene (95). VC serves as an essential substrate for ACCO, playing a dual role by contributing a duplet and acting as a reductant to fully reduce O_2_ to water. VC is also transformed into dehydroascorbate during this process. In the CMD1 reaction, VC not only serves as an electron donor but also contributes a glyceryl moiety to DNA modification, providing a distinct role of VC for non-heme iron-dependent enzymes (Fig. 4) (94).

### *N*^*4*^-methylcytosine

4mC is frequently found as a part of the restriction-modification system in bacterial genome (96). Recently, a new study has demonstrated the existence of 4mC in *Bdelloid rotifers*, a tiny freshwater invertebrate (97). With the 4mC antibody, the authors successfully detected the presence of 4mC in the genomic DNA of the Bdelloid strain *Adineta vaga*. The methylated DNA immunoprecipitation assay further showed a significant enrichment of 4mC in the transposable elements (TEs). To validate these results at single-base resolution, single-molecule real-time (SMRT) sequencing was conducted and 4mC was detected on over 20,000 cytosines, accounting for approximately 0.06% of total cytosines in the genome. The occurrence of 4mC is asymmetric, most prevalently at CpG or CpA dinucleotides. Consistent with methylated DNA immunoprecipitation results, 4mC was found to preferentially target the full-length active TE copies, indicating the association of 4mC with transcriptional repression (97). However, the accurate amount of 4mC in the genome is unclear due to the lack of mass spectrometry data, which could also help to rule out the potential false-positive results from SMRT sequencing.

In addition, the authors identified a 4mC methyltransferase, referred to as N4CMT, which was found exclusively in *A. vaga* and potentially other Bdelloidea, but not in the sister class Monogononta or other eukaryotes (97). Thus, the distribution of N4CMT and 4mC seems to be limited to specific classes, originating from the horizontal transfer of bacterial methyltransferases. The N4CMT contains a catalytic SPPY motif which is commonly found in most bacterial 4mC methyltransferases, distinct from DNMTs for 5mC or 6mA (98). Both the overexpression in bacteria and the *in vitro* protein purification assay have confirmed the methyltransferase activity of N4CMT, notwithstanding all the detection methods depending on the 4mC antibody. In addition, the identification of SETDB1 as a potential 4mC reader protein suggests a link between 4mC and repressive histone mark H3K9me3. Accordingly, 4mC is indicated to function as a potential epigenetic mark, regulating gene expression independently or in cooperation with histone epigenetic marks. Future work is needed to illustrate the distribution and functions of 4mC in diverse eukaryotic organisms. Lastly, as the methylation occurs at the N4 of cytosines, a potential 4mC demethylase might exist to remove the methyl groups directly, which might be similar to the catalytic activity of fat mass and obesity associated and alkB homolog 5 for RNA m6A (99).

### *N*^*4*^-acetyldeoxycytosine

*N*^*4*^-acetylation of cytosines has been reported to occur in RNA (100). Like 5mC and 6mA, which were found to exist both in DNA and RNA, 4acC was recently identified as a type of DNA modification in both plants and mammals (9). In *Arabidopsis*, the abundance of 4acC was determined at 0.1% relative to the total cytosines by mass spectrometry. The authors subsequently performed a 4acC-immunoprecipitation assay and found that 4acC is highly enriched around transcription start sites (TSS) of protein-coding genes. In addition, genes containing 4acC tend to show a higher expression level, reminiscent of the effects of histone acetylation on gene expression (101). 4acC was predominantly found to be enriched in euchromatin regions and exhibit a significantly different distribution pattern from 5mC and 4mC, which are primarily enriched in pericentromeric heterochromatin or TE regions (24, 97). Nevertheless, 4acC has been found to cooperate with 5mC and histone modifications to impact gene expression (9). These data indicated 4acC as an epigenetic mark, but more independent work is needed to further ascertain the distribution of 4acC in other species such as humans and mice (9). Additionally, a comprehensive characterization of the roles and mechanisms of 4acC in transcriptional regulation or as an epigenetic mark is still pending.

The *N*^*4*^-acetylcytosine in RNA was generated by NAT10 and Kre33 in human and yeast, respectively (102, 103). However, the enzymes capable of generating 4acC in DNA have not been reported. Whether DNA acetyltransferases are conserved with NAT10 or Kre33 at the catalytic domain remains unknown. It is interesting to explore the acetyltransferases responsible for 4acC in DNA not only in *Arabidopsis* but also in mammalian cells. In addition, the discovery of the eraser and reader proteins for 4acC is another open question.

### 5hmC-like DNA modification

Although cytosine modifications are pervasive in various organisms, the existence of 5mC in the pathogenic human malaria parasite, *Plasmodium falciparum*, is debatable. In the trial to validate the presence of 5mC and 5hmC in the genome of *P. falciparum*, a previously unanticipated 5hmC-like DNA modification was detected unexpectedly by DNA immunoprecipitation and mass spectrometry (10). The 5hmC-like DNA modification shows similar properties with 5hmC in terms of antibody recognition and bisulfite sequencing. They also share the same qualified transition in the collision-induced dissociation mode of mass spectrometry. But the 5hmC-like modification exhibits a different retention time from 5hmC, indicating that it represents a distinct type of modified nucleoside. Interestingly, the author detected TET-like activity in the nuclear extracts of parasites, despite the absence of TET homologs according to the genomic annotation of *P. falciparum*, suggesting the existence of enzymes containing hydroxylation activity that is different from the classical TET dioxygenases. The 5hmC-like modification is predominantly enriched in gene bodies and is closely associated with gene activation. It would be very interesting to resolve the molecular structure of this DNA modification and to dissect its distribution in various organisms. The similarities and differences between TET proteins and the modifiers for the 5hmC-like modification also deserve further study. It is difficult to conclude whether the 5hmC-like DNA modification is an epigenetic mark or not. Apart from its molecular structure and functions, the identification of the writer protein is a prerequisite for the assessment.

## DNA modifications on adenosines

### *N*^*6*^-methyladenosines

**Figure 5:**
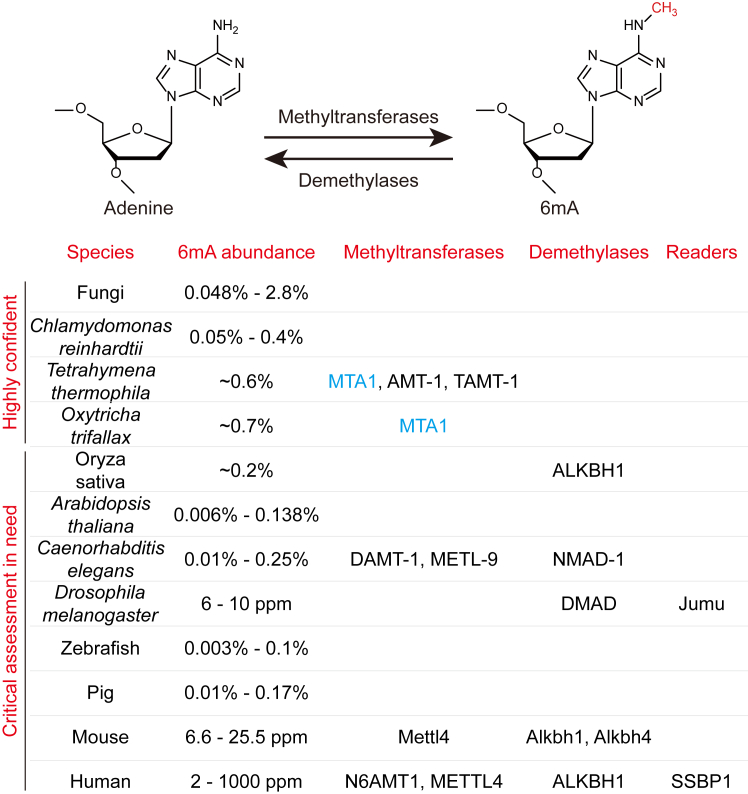
**The abundance of 6mA with putative 6mA methyltransferases and demethylases in various species.** 6mA, *N*^*6*^-methyladenosine.

6mA is another critical component of the restriction-modification system in bacteria and was previously thought to exist exclusively in prokaryotes (96). In contrast, adenine *N*^*6*^-methylation is abundant in mammalian mRNA (m6A) and plays key roles in regulating mRNA stability, translation efficiency, and nuclear exportation (104). However, the existence of adenine methylation in eukaryotic DNA remains unclear. In 2015, three back-to-back studies simultaneously reported the existence of 6mA in eukaryotic DNA in three different organisms: *C. reinhardtii*, *Caenorhabditis elegans*, and *Drosophila*, shedding new light on the discovery of novel DNA modifications (105, 106, 107). Interestingly, the 5mC level in *C. elegans* and *Drosophila* is extremely low, highlighting the importance of 6mA in both organisms (28, 108). 6mA has been subsequently detected in a wide range of eukaryotes, including various fungi (109), plants (110, 111), zebrafish, pigs (112), and even mice (113) and humans (114, 115) (Fig. 5).

However, the abundance of 6mA varies dramatically and the functions of 6mA differ in distinct organisms. Among these eukaryotes, 6mA marks up to 2.8% of total adenosines in the fungi, much higher than in other eukaryotes reported. 6mA was found to symmetrically occur at ApT dinucleotides surrounding the TSS sites. The distribution of 6mA is inversely correlated with that of 5mC but is positively correlated with gene expression (109). 6mA is also abundant in the genome of *C. reinhardtii*, constituting approximately 0.4% of total adenines in DNA. In *C. reinhardtii*, 6mA is dynamically regulated, indicating the potential involvement of 6mA in the regulation of cell cycles. Similar to that in fungi, 6mA predominantly occurs at ApT dinucleotides around TSS with a bimodal distribution, with a specific enrichment at actively transcribed genes in *C. reinhardtii* (105). A similar pattern of 6mA in the ApT motif was observed in *Tetrahymena*, with specific enrichment of 6mA in the linker DNA regions flanking by well-positioned nucleosomes and/or H2A.Z-containing nucleosomes. However, 6mA was found to be uncorrelated with highly expressed genes in *Tetrahymena* (116). Further studies confirmed the enrichment of 6mA in the VATB motif (V = A, C, or G; B = C, G, or T) both in *C. reinhardtii* and *Trypanosoma thermophila* (117). In *Drosophila*, a putative 6mA demethylase DNA 6mA demethylase (DMAD) was identified to catalyze the demethylation of 6mA both *in vitro* and *in vivo*, and was shown to be essential for the embryonic development of *Drosophila*. The mechanistic study showed that DMAD-mediated 6mA removal is correlated with transposon suppression and the loss of DMAD might result in genome instability and embryonic lethality (107). Although the 6mA level in *Drosophila* is relatively low (less than 10 ppm), a Fox-family protein Jumu was found to bind with 6mA to regulate the proper expression of *zelda*, which is critical for the maternal-to-zygotic transition (118). Moreover, the accumulation of 6mA by DMAD depletion was shown to coordinate with the Polycomb complex in transcriptional repression in neurons, indicating a more complicated role of 6mA in gene expression (119). In *C. elegans*, the 6mA demethylase NMAD, as well as a putative 6mA methyltransferase Damt-1 has been documented (106). However, due to the inability to purify the Damt-1 protein from bacteria or insect cells, it is uncertain whether it contains authentic 6mA methyltransferase activity or merely acts as a 6mA regulator. Recently, another 6mA methyltransferase METL-9 was identified in *C. elegans*. The dynamic change of 6mA was observed in response to pathogenic infection and METL-9 was shown to be essential for innate immunity of *C. elegans* (120). Moreover, 6mA was found to be a transgenerational epigenetic mark to modulate mitochondrial stress (121) and also found to mark the TEs in aging control (122). In vertebrates, 6mA was found to accumulate during early embryogenesis in zebrafish and pigs but decreased to the background level upon embryonic development, suggesting a periodic regulatory role of 6mA in certain species (112).

Although the writer and eraser proteins are not completely understood in many organisms, it is unequivocal that 6mA is present in eukaryotes and serves as a stable epigenetic mark in some species, such as *C. reinhardtii* and *T. thermophila*. However, the major dispute within this field originates from the presence of 6mA in mammalian cells. With SMRT of chromatin immunoprecipitation–enriched DNA sequencing, 6mA was detected at H2A.X deposited regions in mouse ES cells, at a level 4-fold higher than basal genomic lines (113). Moreover, the 6mA level was detected to be significantly elevated in mouse brains upon stress (123), while the leukocyte 6mA level was shown to be significantly lower in patients with chronic kidney disease or related mouse models (124). The existence of 6mA antagonizes the binding of SATB1 to chromatin and is essential for transcriptional regulation during trophoblast development (125). Alkbh1 was identified as 6mA demethylase in mouse ES cells. The manipulation of Alkbh1 in ES cells revealed a positive correlation between 6mA and the silencing of long interspersed nuclear elements 1 transposon and their neighboring genes, opposite to the roles of 6mA in transcriptional activation in lower eukaryotes (113). Another work characterized the Mettl4 and Alkbh4 as the 6mA methyltransferase and demethylase, respectively, proposing the crosstalk between 6mA and repressive histone mark H2A-K119Ub (126). The presence of 6mA in the human genome has also been demonstrated with SMRT sequencing (115). They identified N6AMT1 and ALKBH1 to be 6mA methyltransferase and demethylase and showed the enrichment of 6mA in the coding regions and actively transcribed genes in human cells (115), opposing the reported suppressive role of 6mA in mice. With 6mA-crosslinking-exonuclease-sequencing, 6mA was observed to enrich at active retrotransposons and asymmetrically on the heavy strand of mitochondrial DNA (mtDNA). A mtDNA-binding protein, SSBP1, was shown to recognize 6mA in the regulation of mtDNA replication (127). The mtDNA-enriched 6mA was further confirmed by the other group, suggesting METTL4 as a potential 6mA methyltransferase to regulate mtDNA transcription and replication (128).

However, the existence of 6mA in multicellular eukaryotes was currently challenged by a series of the following studies. In a study conducted by another research group, neither 4mC nor 6mA was detected in mouse ES cells, brains, or livers by mass spectrometry (129). Another study proposed that the potential overestimation of 6mA would result from the contamination of bacterial enzymes used for DNA digestion. Comparatively, SMRT sequencing may yield an overestimated false-positive result for 6mA profiling. They also failed to detect 4mC and 6mA in the genomic DNA of various organisms including worms, insects, amphibians, birds, rodents, or primates under normal circumstances (130). More recently, 6mASCOPE, a reformed method based on SMRT sequencing was developed for high-resolution detection of 6mA (117). They found commensal bacteria genome contributes most of 6mA to the detection results of insects and plants. A high abundance of 6mA level was not detected in human cells, even in the glioblastoma stem cells that were previously reported to be enriched with 6mA (114, 117). Several subsequent papers reported that adenine is methylated before misincorporated into the mammalian genome by DNA polymerases (131, 132, 133), arguing against the existence of 6mA methyltransferases. This is consistent with another work reporting that a previously proposed 6mA methyltransferase N6AMT1 is a *bona fide* protein methyltransferase (134). These results strongly resist the presence of 6mA and the role of 6mA as an epigenetic mark in multicellular eukaryotes especially in mammals.

One possible explanation for the inconsistency of the aforementioned studies is the dynamic regulation of 6mA in mammalian cells. The abundance of 6mA varies and depends on the developmental stages or the experienced cellular stress (123). Accordingly, one study found that 6mA accumulated more than 100-fold in glioma stem cells than in normal cells (114), albeit the other work showed that 6mA is significantly lower in gastric and liver cancers (115). 6mA might serve as a hallmark of cytotoxic stresses in glioblastoma cells for increased stemness and proliferative ability although it was postulated as a product misincorporated into DNA (132). 6mA was found to be enriched in mtDNA, 1300-fold higher than genomic DNA (127, 128). The level of 6mA in mitochondria was found to be elevated under hypoxia, supporting the idea that 6mA correlates with stress responses. Nevertheless, the quantitative determination of 6mA in the mammalian genome needs critical reassessment (117).

The best way to resolve the ongoing debate about the roles of 6mA as an epigenetic mark in mammals is to find out the 6mA methyltransferases and demethylases (Fig. 5). As mentioned before, several 6mA demethylases such as DMAD, Alkbh1, and Alkbh4 have been proposed with their activity determined by *in vitro* or *in vivo* assays. Chemically, the demethylation from N6 of adenine is more efficient compared to that of 5mC, due to the relatively lower stability of C-N bonds. Notably, although 6mA was thought to be randomly incorporated into the genome of Hydractinia, Alkbh1 can still function as a 6mA eraser to facilitate zygotic genome activation (135). Interestingly, a TET homolog from mushrooms (CcTet) was found to possess the eukaryotic 6mA demethylase activity on duplex DNA (136), in addition to its primary function to oxidize 5mC (88). On the contrary, although several proteins have been proposed to be potential 6mA methyltransferases, authentic 6mA methyltransferases were not confirmed until the identification of the MTA1c complex in ciliates (137). MTA1c specifically methylates dsDNA. The complex is composed of two MT-A70 proteins and two homeobox-like DNA-binding proteins (137). MTA1 is the catalytic center, whereas MTA9 and p1 accommodate the substrate DNA, with p2 facilitating the stabilization of MTA1. This might explain why previous work failed to determine the methyltransferase activity with a single enzyme (138). Another work also proposed AMT1, an MT-A70 family protein as 6mA methyltransferase in ciliates (139). Unfortunately, the homologues of MTA1 and MTA9 were not identified in higher organisms such as humans, mice, and *Arabidopsis* (137). In addition, METTL4 and N6AMT1 have been proposed to be putative mammalian 6mA methyltransferases (115, 128, 140). However, the *in vitro* recombinant protein assay is still needed to confirm the activity. The RNA m6A methyltransferase complex METTL3/METTL14 was found to utilize dsDNA as substrates to generate 6mA *in vitro*, and METTL3 shares a conserved DPPW motif with MTA1 (141). Further investigation is required to determine if this complex shows similar activity *in vivo*. However, another study suggested that 6mA in the mammalian genome was introduced by the RNA m6A machinery *via* a nontargeted mechanism (142). Due to the relatively low abundance of 6mA and potentially weak activity of the methyltransferases in mammals, the identification of 6mA methyltransferases in species other than ciliates, such as green algae, might provide a significant clue for further characterization. More structural data would be helpful to illustrate the presence and conservation of 6mA methyltransferases in mammalian cells. But before the authentic 6mA methyltransferase is identified, it is inconclusive that 6mA exists and functions as an epigenetic mark in mammalian cells.

## Perspectives and challenges

The discovery of novel DNA modifications has been facilitated by rapid-developing approaches exemplified by high-resolution mass spectrometry or next-generation sequencing technologies during the past 15 years. However, it is important to note that certain pitfalls may arise during the detection process (117, 143). The first one is potential contamination from enzymes used in the DNA purification and digestion process. Most of the enzymes are purified from bacteria with prokaryotic genomic DNA, which might lead to false positive results during the detection of 6mA and 4mC, two critical DNA modifications frequently detected in prokaryotes. This situation could be overcome by purifying these enzymes from specific methyltransferase-deficient bacteria strains. Secondly, it is crucial to acknowledge that cultured cells are susceptible to contamination by a trace number of bacteria or mycoplasmas, even if they are not detectable by PCR or other conventional methods. Thirdly, it is inconclusive to determine whether the modification in dsDNA is catalyzed by enzymes or results from mis-incorporated of premodified nucleotides into DNA by polymerases unless a specific writer for this DNA modification is identified. Lastly, various sequencing technologies are used for the profiling of DNA modifications. However, the majority of employed techniques result in an overestimated level of DNA modifications, much higher than the results obtained from mass spectrometry. Consistent data acquired *via* different approaches will help to confirm the presence and abundance of DNA modifications in diverse organisms.

The discovery of novel DNA modifications, especially in eukaryotes, presents a considerable challenge due to the limited abundance in commonly studied model systems. For mammalian cells, it is undoubtedly that the predominant DNA modifications should be 5mC and its derivatives. Further investigation is required to confirm the existence of additional DNA modifications, such as 6mA, 4mC, or other minor DNA modifications, as well as their specific writer proteins. Nevertheless, the pool of lower eukaryotes remains as a treasury for future discovery of novel DNA modifications, since only a limited number of organisms have been well studied with the whole genome sequenced. The diversity of RNA modifications may also offer valuable insight as they share similar properties with DNA modifications.

The discovery of writers and erasers that catalyze DNA modification or demodification is another significant challenge in the field. For instance, the generation of 5mC and its derivatives is extensively studied, but the mechanisms for eliminating the modified moieties remain controversial due to the inertia of C-C bonds. In the case of 6mA, several demethylases have been reported, but the methyltransferase is still less studied in most organisms. Although MTA1 complex has been identified as the 6mA methyltransferase in ciliates, it appears to be absent in mammals and certain other eukaryotes (137), indicating the main catalytic domain of 6mA methyltransferases may not be conserved across all species. Among the proteins that participated in the deposition or removal of DNA modifications, Fe(II)-dependent oxygenases represent an inevitable category, which includes JBP, TET, CMD1, and ALKBH family proteins. All of them use the methyl groups in different nucleosides as substrates, generating various DNA modifications. Exceptionally, the formyl moiety detaches as formaldehyde and results in bona fide demethylation in the ALKBH-mediated reaction (144). The conservation and discrepancies among JBP, TET, and CMD1 might give a hint to further discoveries of novel DNA modifications and modifiers, especially in other undefined organisms (Fig. 3).

To function as a stable epigenetic mark, the DNA modification should be maintained and faithfully transmitted after cell division or passed on to the next generation. This requires not only the DNA modification writer proteins. However, there remains a knowledge gap concerning the exact mechanisms by which DNA is *de novo* modified and maintained accurately at specific sites. The crosstalk between histone modifications, RNA modifications, and DNA modifications has been reported (145, 146), which holds the potential to throw light on the study of the accuracy and complexity of DNA modifications as epigenetic marks in the future.

## Conflict of interest

The authors declare that they have no conflicts of interest with the contents of this article.
